# Analyzing the Impact of the Highest Expressed Epstein–Barr Virus-Encoded microRNAs on the Host Cell Transcriptome

**DOI:** 10.3390/ijms25147838

**Published:** 2024-07-17

**Authors:** Tim Hohmann, Urszula Hohmann, Faramarz Dehghani, Olaf Grisk, Simon Jasinski-Bergner

**Affiliations:** 1 Department of Anatomy and Cell Biology, Medical Faculty, Martin Luther University Halle-Wittenberg, Grosse Steinstrasse 52, 06108 Halle (Saale), Germany; tim.hohmann@uk-halle.de (T.H.); urszula.hohmann@uk-halle.de (U.H.); faramarz.dehghani@medizin.uni-halle.de (F.D.); 2 Institute of Physiology, Brandenburg Medical School (MHB), Theodor Fontane, Hochstraße 29, Haus 11, 2.OG, 14770 Brandenburg an der Havel, Germany; olaf.grisk@mhb-fontane.de; 3 Institute for Translational Immunology, Brandenburg Medical School (MHB), Theodor Fontane, 14770 Brandenburg an der Havel, Germany

**Keywords:** EBV, microRNA, transcriptome, RNA sequencing, immunology, immune evasion

## Abstract

The Epstein–Barr virus (EBV) has a very high prevalence (>90% in adults), establishes a lifelong latency after primary infection, and exerts an oncogenic potential. This dsDNA virus encodes for various molecules, including microRNAs (miRs), which can be detected in the latent and lytic phases with different expression levels and affect, among others, immune evasion and malignant transformation. In this study, the different EBV miRs are quantified in EBV-positive lymphomas, and the impact on the host cell transcriptome of the most abundant EBV miRs will be analyzed using comparative RNA sequencing analyses. The EBV miRs ebv-miR-BART1, -BART4, -BART17, and -BHRF1-1 were most highly expressed, and their selective overexpression in EBV-negative human cells resulted in a large number of statistically significantly down- and up-regulated host cell genes. Functional analyses showed that these dysregulated target genes are involved in important cellular processes, including growth factor pathways such as WNT, EGF, FGF, and PDGF, as well as cellular processes such as apoptosis regulation and inflammation. Individual differences were observed between these four analyzed EBV miRs. In particular, ebv-miR-BHRF1-1 appears to be more important for malignant transformation and immune evasion than the other EBV miRs.

## 1. Introduction

The human Epstein–Barr virus (EBV) is not only the causative agent of infectious mononucleosis (IM), but together with Kaposi’s sarcoma herpesvirus, they represent a subgroup of the γ-herpesviruses, which has oncogenic potential and can contribute to the malignant transformation of infected host cells [1]. In fact, the following malignancies are associated with EBV infections: Burkitt’s lymphoma (BL), NK/T-cell lymphoma, classical Hodgkin’s lymphoma, gastric carcinoma (GC), and nasopharyngeal carcinoma (NPC) [2].

EBV is a double-stranded (ds) DNA virus whose genome comprises approximately 172 kbp [3]. The global EBV prevalence is around 90% in adults; this is due to its ability to establish a lifelong latency after the primary infection, which usually occurs during childhood or adolescence and is indeed a characteristic feature of the herpesviruses [3,4,5]. EBV most efficiently infects and transforms resting B cells in vitro because, in the viral entry mechanism, viral envelope proteins interact with CD21, CD35, and HLA class II [6,7]. In fact, during IM, an estimated 1 in 10^4^ circulating B cells is EBV-positive, while in adults with latent EBV infection, only 1–50 out of 10^6^ B cells are EBV-positive [8].

Despite EBV hiding only in a small number of infected host cells, it avoids detection and elimination by immune effector cells using molecular mechanisms involving a variety of EBV-encoded molecules, which range from coding and non-coding transcripts to peptides and even proteins [9].

Interestingly, CD21-negative cells, including T and NK cells, can also be infected by EBV and are linked to extranodal NK/T-cell lymphoma-nasal type, aggressive NK-cell leukemia (ANKL), and EBV-T/NK-lymphoproliferative diseases [10]. Current research focuses on the identification of additional receptors that are involved in the viral entry mechanism, such as ephrin receptor tyrosine kinase A2 for EBV infection of epithelial cells [11], a fact that might be relevant for EBV-associated NPCs and GCs. However, the specific T- or NK-cell receptors for EBV entry are still unknown.

The viral gene expression strongly differs between the latent and lytic phases and can even be further divided into subgroups based on the expression of individual genes [12]. In infected and circulating memory B cells in vivo, there is latency 0 phase, in which only non-coding transcripts can be detected, while in the latency 1 phase, typically observed in BL, EBNA1 expression, along with the non-coding viral transcripts, is observed. This is followed by the latency 2 phase, characteristic for cHL and NPC, which exhibits an additional expression of LMP1 and LMP2, while in the latency 3 phase of B cell transformation, EBNA2, EBNA-LP, BHRF1 and the three latent membrane proteins (LMP) 1, 2A, and 2B, in addition to the non-coding transcripts, can be measured [12].

The expression of non-coding transcripts occurs in all phases of the viral life cycle. Particularly in the phases of latency, one must speculate about an important contribution of these non-coding transcripts and their role for immune evasion as well as for the molecular biological processes of malignant transformation, which is all the more fatal in an already established immune-suppressed environment. Current literature shows the importance of the two long non-coding RNAs EBER1 and EBER2 in enhancing resistance to apoptosis (including IFN-γ-induced apoptosis [13,14]), the increase in anti-inflammatory cytokines like IL-10 [15], and the induction of growth factors like IGF-1 [16].

Apart from these long non-coding transcripts, EBV also encodes different microRNAs (miRs), which are located in three clusters (BHRF1 cluster, BART cluster 1, BART cluster 2) within the EBV genome. miRs are small 18–22 nt long single-stranded RNAs with a binding sequence specifically for their target (host) mRNAs (mostly within the 3′-UTR leading to their translational repression and mRNA decay [17]). It is estimated that up to 60% of human mRNAs are directly or indirectly regulated by miRs as an additional instance of posttranscriptional gene regulation [18]. Each miR has the potential to target a huge number of different mRNAs, and vice versa, one mRNA can be regulated by multiple miRs [18]. It is thought that most miR–mRNA interactions involve perfect complementary binding by the so-called seed region (second to eighth nt of the miR), but miR–mRNA binding without perfect seed complementarity has also been reported by the existence of seed–distal complementarity [19,20]. However, within the binding site between miRs and mRNA, there might be mismatch pairings, bulges, and wobble base pairs, reducing the accuracy of in silico-based prediction tools to false-positive rates of 20–40% [21].

The many different human miRs can even be grouped according to the functions of their target mRNAs, so one can distinguish between oncogenic miRs, e.g., hsa-miR-21-5p, and anti-tumoral miRs, such as hsa-miR-34A-5p [22,23,24]. Interestingly, recent research has identified many EBV-encoded miRs directly contributing to the immune evasion of infected host cells. The effects of EBV-encoded miRs are diverse and range from reducing apoptosis sensitivity by targeting PUMA, disrupting antigen processing and presentation (ebv-miR-BHRF1-3; ebv-miR-BART17), and down-regulating pro-inflammatory or even anti-viral cytokines IFNL3 [25,26,27].

Due to the fact that different EBV-encoded miRs regulate different human target genes, e.g., directly or indirectly down-regulation or indirectly up-regulation, the hypothesis should be investigated whether the EBV-encoded miRs that are most highly expressed during latency act on tumor-biologically relevant pathways, and as a consequence, how are they involved in important processes like immune evasion and malignant transformation?

In this study, the most highly expressed EBV-encoded microRNAs were first identified by qPCR. Subsequently, respective expression vectors were cloned and functionally validated, followed by transfection into EBV-negative immune-competent human cells, followed by RNA sequencing-based transcriptome analyses for the identification of regulated target genes as well as pathways with relevance for immune evasion.

## 2. Results

**Figure 1:**
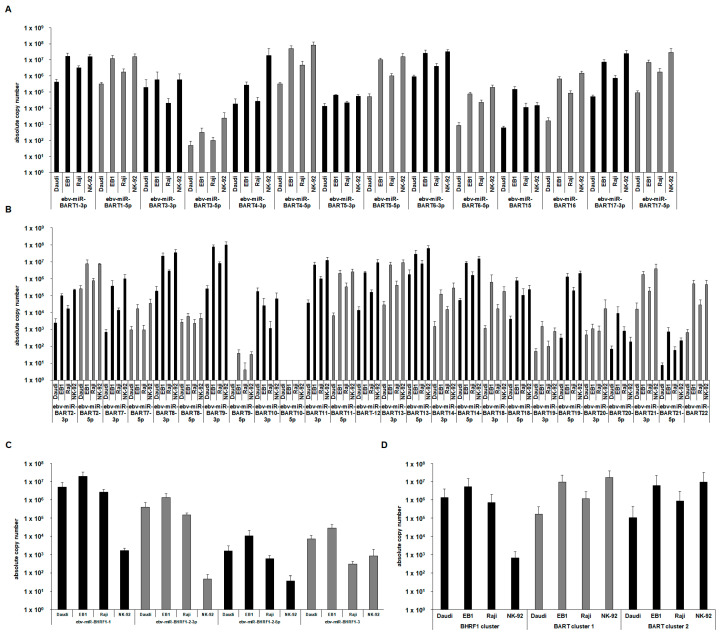
Determining absolute copy numbers by qPCR of the EBV miRs expressed as means of six biological replicates from each EBV-positive cell line for the BART1 cluster (**A**), BART2 cluster (**B**), BHRF cluster (**C**), and summed expression values for the combined comparison of the individual EBV miR clusters of the respective cell lines (**D**); (*n* = 6 biological replicates).

Initially, the EBV-encoded miRs were validated in four EBV-positive lymphoma cell lines using qPCR. The three B-cell lymphoma cell lines, Raji, EB1, and Daudi, as well as the NK-cell lymphoma cell line NK92, were used for this purpose. To ensure optimal comparability, the cell culture of these cell lines, the RNA extraction, template-specific cDNA synthesis for the quantification of a respective miR, and quantification by qPCR were carried out in parallel and under the same conditions from six biological replicates of each. All of the EBV-encoded miRs, which are listed in the online database www.miRbase.org [28], were quantified using qPCR. For a better comparison of these different cell lines, absolute copy numbers were determined. The EBV miR genes within the EBV genome are located in clusters [29]. There are 14 EBV miRs in the BART1 miR cluster and another 26 in the BART2 cluster. In addition, the four miRs—ebv-miR-BHRF1-1, ebv-miR-BHRF2-5p, ebv-miR-BHRF2-3p, and ebv-miR-BHRF1-3—are encoded in the BHRF cluster (Figure 1A–C).

Interestingly, there were only a few cases where differences between the four different EBV-positive cell lines for the expression of a single miR could be detected. In the BART1 cluster, the cell line NK92 showed much higher expression of ebv-miR-BART4-3p than the other cell lines (Figure 1A), while the Daudi cell line showed the lowest expression for most of the different miRs of this BART1 cluster, sometimes reduced by up to a factor of 100.

In accordance with that, it was found in the BART2 cluster that the Daudi cell line often exerted a reduced expression of several EBV miRs, frequently around a factor of ten. In fact, in the case of ebv-miR-BART9-5p, which was only very weakly detectable in all cell lines, it was shown that this miR was completely undetectable in Daudi cells. Surprisingly, the miR ebv-miR-BART10-5p could not be detected in any of the cell lines used, which was reproducible (Figure 1B). When quantifying the four EBV miRs of the BHRF cluster, it was shown that the NK92 cells had reduced expression for all four BHRF miRs, in some cases reduced by three orders of magnitude (Figure 1C). For a better analysis of these findings, the respective miR expression of each EBV miR cluster was summed up for the corresponding cell lines and compared. In fact, the Daudi cell line shows a clearly reduced expression of EBV miRs in the BART1 cluster and in the BART2 cluster (about a factor of ten), while the other three cell lines show comparable expression values. Instead, in the more distant BHRF cluster, the cell line NK92 shows an extremely reduced expression of the local EBV miRs (by three powers of ten). The other three cell lines have comparable expression levels in the BHRF cluster (Figure 1D). According to the project hypothesis, the most abundant EBV miRs should be identified. It was taken into account that, if possible, both miRs of a stem-loop (-5p and -3p) are highly expressed. This led to the identification of ebv-miR-BART1, ebv-miR-BART4, ebv-miR-BART17, and ebv-miR-BHRF-1-1 as the four most abundant EBV miRs, which were selected for further analyses.

Respective overexpression vectors were cloned and functionally validated according to Jasinski-Bergner et al., 2023 [27]. For this purpose, the sequenced plasmid constructs and the control plasmid were transiently transfected into EBV-negative HEK293T cells. The hsa-miR-541 was selected as the appropriate control miR for the overexpression studies as well as for the transcriptome analyses because hsa-miR-541 is naturally not expressed in HEK293T, reflecting the situation of the EBV miR overexpression in the EBV-negative HEK293T cells.

**Figure 2:**
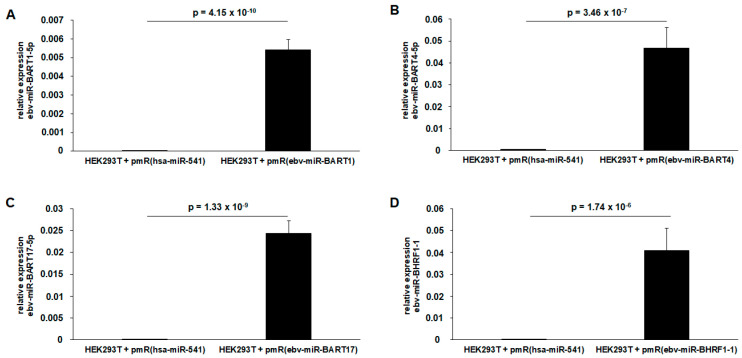
Functional validation by qPCR of the cloned EBV miR expression plasmids in transiently transfected EBV-negative HEK293T cells. For this quantification of miRs within a single cell line, the determination of relative copy numbers was performed for overexpression of ebv-miR-BART1 (**A**), ebv-miR-BART4 (**B**), ebv-miR-BART17 (**C**), and ebv-miR-BHRF (**D**) (*n* = 3 biological replicates; p, two-tailed Student’s t-distribution).

The large T antigen of the HEK293T cells, as well as the SV40 origin of replication of the employed plasmids in combination with the highly transfectable properties of this cell line, offers the best conditions to reach the high expression levels of virally encoded molecules for further studies, strongly highlighting the HEK293T model system as a standard in such virological studies [30]. In fact, after 72 h of transient transfection, RNA was extracted and quantified by qPCR. There was an extremely strong, statistically significant overexpression of the corresponding EBV miRs in the respective HEK293T transfectants compared to the overexpression of the control miR (hsa-miR-541). Examples of this are ebv-miR-BART1-5p (*p* = 4.15 × 10^−10^), ebv-miR-BART4-5p (*p* = 3.46 × 10^−7^), ebv-miR-BART17-5p (*p* = 1.33 × 10^−9^), and ebv-miR-BHRF1-1 (*p* = 1.74 × 10^−6^), shown in Figure 2A–D, which were quantified by determining relative copy numbers. In this homogeneous HEK293T transfection system, the determination of relative copy numbers took place by using the endogenous human miR hsa-miR-3960. As already described in Jasinski-Bergner et al., 2022 [1], this offers a variety of advantages that can be used in the quantification of these miRs, which is opposite to the quantification of the miRs in various EBV-positive B and NK cell lines, and which is why absolute copy numbers were previously determined for better comparability.

After the successful functional validation of the EBV miR-overexpressing plasmids by qPCR, the respective HEK293T transfectants were used for transcriptome analyses. Three biological replicates of each were applied using RNA sequencing in the core unit “DNA technologies” (PD Dr. Knut Krohn, University of Leipzig, Medical Faculty, Germany).

**Figure 3:**
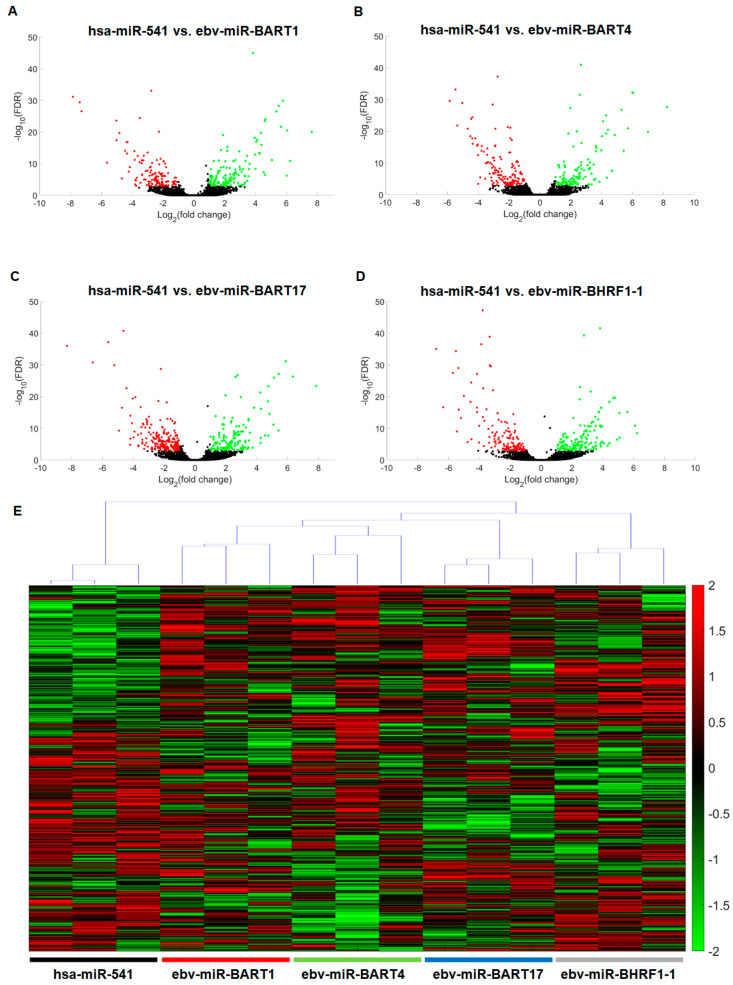
The results of the transcriptome analyses of EBV-negative HEK293T transiently transfected with EBV miR expression plasmids for overexpression of ebv-miR-BART1 (**A**), ebv-miR-BART4 (**B**), ebv-miR-BART17 (**C**), and ebv-miR-BHRF1-1 (**D**) were visualized as volcano plots with statistically significantly down-regulated or up-regulated genes in red or green. The overexpression of hsa-miR-541, which is also not naturally expressed in HEK293T cells, served as a control. As a consequence of previous cluster analyses (see Supplemental Figures S1 and S2), a hierarchical clustering analysis (**E**) was performed. All genes, ordered as in the heatmap, and their respective scores are listed in Supplemental Table S2.

To identify and visualize statistically significantly up- or down-regulated genes, corresponding volcano plots were generated for the overexpression of every respective EBV miR (Figure 3A–D). It was shown that when overexpressing ebv-miR-BART1, 160 or 137 genes were statistically significantly up- or down-regulated. The same analysis was done when overexpressing ebv-miR-BART4 (up *n* = 139, down *n* = 165), overexpressing ebv-miR-BART17 (up *n* = 167, down *n* = 225), and overexpressing ebv-miR-BHRF1-1 (up *n* = 167, down *n* = 178). The gene names and expression values can be found in Supplementary Table S1. The total number of unique genes in this list was 933. After preprocessing genes for cluster analysis to remove genes with low variability that likely do not contribute to a gene signature, this list was reduced to 528 genes. Clustering analysis then showed that the samples were best grouped in five clusters, corresponding to the four ebv-miR groups and the control, respectively (Supplementary Figures S1 and S2, Supplementary Table S2). The hierarchy of the clusters demonstrated that the gene expression patterns of the three biological replicates of the EBV miRs have a higher self-similarity among each other than to the human control miR transfectants (Figure 3E).

**Figure 4:**
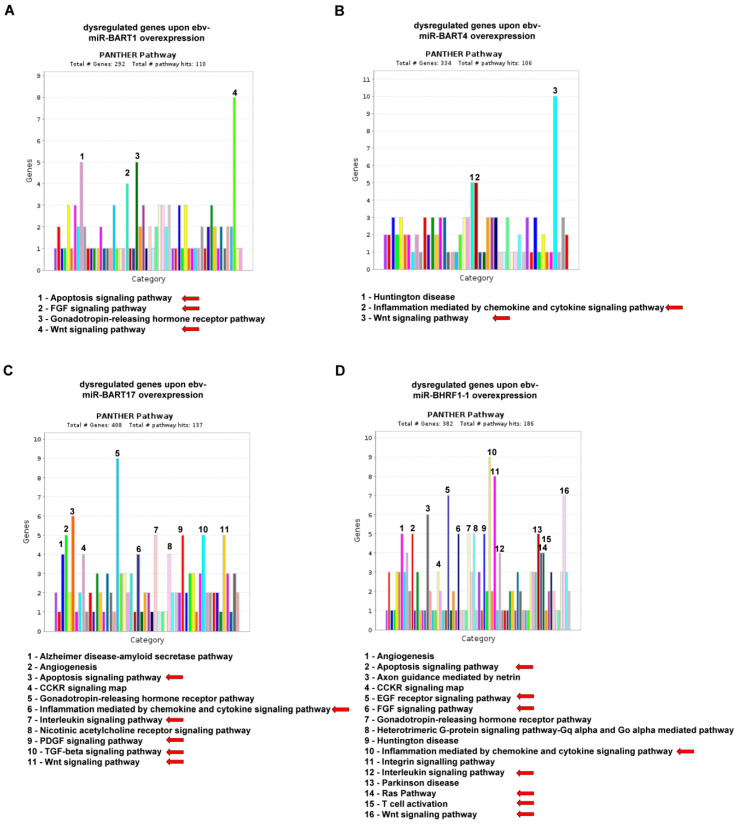
For the statistically significantly down-regulated or up-regulated genes upon the overexpression of ebv-miR-BART1 (**A**), ebv-miR-BART4 (**B**), ebv-miR-BART17 (**C**), and ebv-miR-BHRF1-1 (**D**), functional enrichment of differentially expressed genes was performed using PANTHER (https://pantherdb.org/webservices/go/overrep.jsp [31,32]; accessed on 1 April 2024). The pathways with the most dysregulated genes were marked with numbers and are listed by name below the bar chart. Pathways relevant to tumor biology or tumor immunology are marked with a red arrow.

In order to get an impression of which target genes and which biological processes are dysregulated by the overexpression of the most abundant EBV miRs, all statistically significantly dysregulated genes were used for the annotation clustering. This was carried out separately for all four different EBV miR-overexpressing transfectants using the database https://pantherdb.org/ [31,32] (accessed on 1 April 2024), which groups genes according to their functionality (GO terms) and pathway analyses, among other things. The results are shown in Figure 4A–D, whereby functional groups with a particularly large number of dysregulated genes have been marked with an arrow. All four different EBV miRs dysregulate growth factor pathways such as WNT, EGF, FGF, and PDGF, but to a different extent. Furthermore, certain cell biological processes, such as apoptosis and inflammation, were also affected. This was particularly pronounced when overexpressing ebv-miR-BHRF1-1. Although the absolute number of up- and down-regulated genes was similar to that of the other EBV-miR-overexpressing transfectants, ebv-miR-BHRF1-1 overexpression revealed a very strong dysregulation of growth factors, apoptosis regulation, and inflammation-relevant pathways, as well as RAS, which is also a proto-oncogene (Figure 4D).

To estimate the extent to which the results of such isolated EBV-miR expression experiments in EBV non-relevant HEK293T cells can be translated to real in vivo EBV infections, comparisons were carried out. For this purpose, cDNA microarray data sets available at the R2 microarray database (http://r2.amc.nl; accessed on 1 May 2024) of EBV-positive B-cell lymphomas [33] and flow cytometry-sorted CD19-positive healthy EBV-negative B cells [34] were carried out. As the database samples were generated using microarrays, some candidate genes were not present in those samples, reducing the number of target genes that can be analyzed from 933 differentially expressed genes to 573, corresponding to 209 up-regulated and 364 down-regulated genes (Supplementary Table S3). While a single EBV miR was overexpressed in the HEK293T cells for a temporarily limited period of 72 h in the in vitro model, the EBV-infected and malignant transformed lymphoma cells possess the full spectrum of EBV miRs (and other EBV-encoded molecules) and altered expression levels as a result of the malignant transformation itself, as well as possibly existing individual counter-regulatory mechanisms of the host cell, representing a strong increase in the complexity of this in vivo situation. However, it was found that of the statistically significantly down-regulated genes of all four EBV miRs examined, 23% (83 of 364 genes) were actually still statistically significantly down-regulated in the in vivo EBV-positive B-cell lymphoma cells, and vice versa, 27% (57 of 209 genes) of the statistically significantly up-regulated genes were also statistically significantly up-regulated in the EBV-positive B-cell lymphomas, corresponding to an enrichment factor of 14.7 or 30.7, respectively.

## 3. Materials and Methods

### 3.1. Cell Culture, Cell Transfection, Cell Harvest, Flow Cytometry

All human cell lines used in this study were purchased from the American Type Culture Collection (ATCC, Manassas, VA, USA). HEK293T cells were cultured in DMEM (Thermo Fisher, Waltham, MA, USA), and the B/NK cells were cultured in RPMI-1640 medium (Thermo Fisher). Both media were supplemented with 10% (*v*/*v*) fetal bovine serum (Thermo Fisher), 1% (*v*/*v*) Penicillin-Streptomycin (Thermo Fisher), 2 mM sodium pyruvate (Thermo Fisher), and 1-fold GlutaMax (ThermoFisher). The cells were incubated at 37 °C and 5% (*v*/*v*) CO_2_ using the standard water pan in an incubator (Binder, Tuttlingen, Germany).

HEK293T cells were chemically transfected with DNA plasmids for miR overexpression, including a control miR plasmid as control, with TurboFect transfection reagent (Thermo Fisher) according to the manufacturer’s instructions. The transfected cells were harvested 72 h after transfection. Transfection efficiency was controlled by the determination of the mCherry fluorescence on a FACSCelesta (Becton Dickinson, Franklin Lakes, NJ, USA).

### 3.2. RNA Extraction, cDNA Synthesis, qPCR, and Generation of the miR Expression Vectors

The NucleoSpin miRNA extraction kit (Macherey-Nagel, Düren, Germany) was used for the later microRNA analyses, while the NucleoSpin RNA extraction kit (Machery-Nagel) was applied for the RNA extraction prior to the RNA sequencing analyses, in both cases according to the manufacturer’s instructions.

For the miR quantification, six biological replicates of each analyzed cell line were employed for template-specific cDNA synthesis after the stem-loop method published by Chen et al., 2005 [35]. This template-specific cDNA synthesis was performed separately for each analyzed miR, including all of the ebv-miRs listed at www.mirbase.org [36] (accessed on 1 January 2024), by using 1000 ng of purified RNA and a miR-specific stem-loop primer, which are listed in Supplemental Table S4.

Absolute copy numbers were determined for the quantification of the EBV miRs within the different EBV-positive cell lines by qPCR (GoTaq, Promega, Madison, WI, USA). To validate the functionality of the EBV miR overexpressing plasmids in EBV-negative HEK293T cells, relative copy numbers were determined by using the highly expressed hsa-miR-3960 as an endogenous reference gene.

The EBV miR overexpression plasmids were generated by amplification of the respective inserts by PCR using cloning primers listed in Supplemental Table S4 and applying the restriction enzymes EcoRI, BamHI, and XhoI, as well as the T4 DNA ligase (Thermo Fisher) and the pmR-mCherry vector (Takara Bio, Kusatsu, Japan).

### 3.3. RNA Sequencing Analyses

The transcriptome analyses were performed at the Core Unit “DNA technologies” (PD Dr. Knut Krohn, University of Leipzig, Medical Faculty, Germany). Therefore, the quality control of total RNA was checked with the Fragment Analyzer 5200 (Agilent, Santa Clara, CA, USA) using the High Sensitivity RNA quantification kit and Fragment Analyzer Controller Software (Agilent v3.1.0.12). Random primed library preparation was started with 150 ng of total RNA using the Watchmaker RNA library prep kit with Polaris depletion (Watchmaker Genomics, Boulder, CO, USA) according to the instructions of the manufacturer. The barcoded libraries were purified and quantified using Qubit Fluorometric Quantification (Thermo Fisher). The size distribution of the library DNA was analyzed again using the Fragment Analyzer 5200 (Agilent). Sequencing of 2 × 150 bp was performed with an Illumina NovaSeq sequencer (Illumina, San Diego, CA, USA) at the sequencing core facility of the Faculty of Medicine (University Leipzig) according to the instructions of the manufacturer. After demultiplexing with bcl2fastq software (Illumina, v2.20) and polishing using FASTP [37,38], reads were mapped against the human reference genome (hg38) using HISAT2 [39]. Stringtie and the R package Ballgown [40] were employed for transcript quantification with DESeq2 [41] normalization.

### 3.4. Analysis of RNA-Seq Data

Raw counts were processed and analyzed using DESeq2 to obtain differentially expressed genes [41]. Genes were considered differentially expressed if the fold change was larger than 2 or smaller than 0.5 and the estimated false detection rate was lower than 0.05. Functional enrichment of differentially expressed genes was analyzed using PANTHER (https://pantherdb.org/webservices/go/overrep.jsp [31,32], accessed on 1 April 2024).

Cell lines were clustered by gene signature using an approach described elsewhere [42]. Briefly, differentially expressed genes of each sample were log-transformed and median-centered. Afterwards, the standard deviation for each individual gene across all samples was set to one, and genes with a median absolute deviation smaller than 0.5 were discarded. To cluster the samples, consensus clustering using hierarchical clustering with agglomerative average linkage was used. The 1-Pearson Correlation Coefficient served as the distance metric. Consensus clustering was performed 1000 times with a sub-sampling ratio of 0.8. The exact number of clusters was determined from the confusion matrix for each cluster number and the cumulative density function of the consensus matrix for each possible cluster number.

To verify the previously identified differentially expressed genes, microarray data samples of pure B-cell subsets (NCBI: GSE12366) were compared to lymphoma cells (NCBI: GSE4086). For comparing both microarray data sets, data was first quantile normalized [42,43], followed by joint removal of low-expressing and low-variance genes (both: 10 percentile). Differential gene expression was calculated using permutation T-tests on the log-transformed data, followed by false discovery rate calculation [44,45]. Genes were considered significantly deregulated if the false discovery rate was smaller than 0.05 and the fold change expression was larger than 2 or smaller than 0.5. Afterwards, the number of overlapping genes between those samples and the miR-transfected cells was calculated.

## 4. Conclusions

Indeed, the data show that there are significant differences in the expression of the respective EBV miRs. In addition, there are corresponding expression differences between the cell lines. After EBV entry into the host cell, the linear viral DNA is circularized and chromatinized to form an episome in the nucleus [46], and during mitosis, the viral EBNA1 binds to the EBV origin of plasmid replication (oriP) to initiate replication of the viral DNA [47]. Furthermore, EBNA1 also relocates the episomes to the host cell chromosomes, which allows episomes to segregate into daughter cells with the host chromosome, maintaining a stable episome copy number [48,49]. The observed differences in EBV miR expression between the applied EBV-positive cell lines could, therefore, be due to different numbers of viral episome copies or to the general ability of the cell lines to process miRs per se, although both points cannot explain the observed differences in the expression of individual EBV miRs or selectively reduced expression values of individual EBV miR clusters. Indeed, during latency, many viral genes are epigenetically repressed by cellular chromatin constituents and DNA methylation, while in infectious particles, viral DNA is free of histones and lacks methylated cytosine residues, which are lost during lytic DNA amplification [50]. So, the observed differently expressed EBV miR genes may also be due to different EBV life phases, e.g., lytic or late (sub)phases of the applied cell lines. At least EB1 cells are known to express the immediate-early protein BZLF-1, marking this cell line in a different life cycle phase than the other EBV-positive cell lines. However, the different ebv-miR expression profiles between the analyzed cell lines could also indicate entity-specific differences, especially the differences between the NK-cell lymphoma cell line NK92 and the B-cell lymphomas.

The combination of the generated EBV miR expression plasmids and the transient transfection in HEK293T cells generated an expression level of the EBV miRs that was comparable to the respective expression level in the EBV-positive cell lines. This fulfills an important prerequisite for the following transcriptome analyses, which were performed using RNA sequencing and bypassing cDNA microarrays, which are limited by the number of present probes. In addition, the applied procedure of separated EBV miR overexpression allows for a precise assessment of whether the individual EBV miRs play different roles in the processes of (tumor) immune evasion and malignant transformation, be it changes in metabolism, proliferation, migration, apoptosis regulation, antigen presentation, expression of genes involved in the innate or adaptive immune system, or even genetic stability, e.g., regulation of tumor suppressor genes, etc. This led to the observation that abundant EBV miRs actually intervene in tumor-biological and immunologically relevant pathways of the host cell. In doing so, they directly regulate different target genes in a sequence-specific but also in an indirect manner, and together, they contribute to the success of immune evasion and malignant transformation. This study also showed that the ebv-miR-BHRF1-1 appears to have a stronger influence on these tumor-relevant processes than the other three EBV miRs, which are also similarly expressed. Notably, genes affected by EBV miR overexpression are both supposed to be involved in tumor initiation and spreading. For example, the genes BCL9L or SKI—found to be up-regulated in vivo and in vitro—are potentially involved in tumor growth and initiation via modulation of the TGFβ (SKI) or WNT-β-catenin (BCL9L) pathway [51,52,53]. In addition, genes related to tumor spreading, cell migration, and cytoskeletal reorganization were also up-regulated in vitro and in vivo, such as CDC42, DNMBP, CAPG, NRAS, BCL9L, etc. [54,55,56,57,58]. Interestingly, DNMBP is a guanine exchange factor (GEF) regulating CDC42 [55,56], strengthening the hypothesis about an important role of EBV miRs in cancer. Thus, it appears plausible that the EBV-induced miR may potentially even be involved in multiple stages of cancer.

For the interpretation of the in vivo cDNA microarray data sets, it is important to note that the EBV-positive B-cell lymphomas not only express the most abundant EBV miRs simultaneously but also the entirety of all EBV miRs, as well as other EBV-encoded molecules, such as the two long non-coding RNAs EBER1 and EBER2 and other EBV-encoded molecules, including viral peptides and viral proteins simultaneously and together. It should also be noted that the human B-cell lymphomas have already completed the malignant transformation, which also has a changing and, thus, limiting influence on their transcriptome when compared to the dysregulated genes of the HEK293T transfection models. Nevertheless, these obtained similarities of statistically significantly dysregulated genes show that the approach is suitable for identifying and characterizing abundant EBV miRs in the process of (tumor) immune evasion and malignant transformation of infected human host cells and that this regulatory potential really exists, even in single isolated EBV miRs (in vitro), which in vivo is in combination with all EBV miRs and all EBV-encoded molecules, together developing a corresponding potential for (tumor) immune evasion and malignant transformation. These abundant EBV miRs could thus serve as prognostic markers but also as target structures.

An important limitation must be noticed when considering these data. As already mentioned in the introduction, EBV infects not only lymphocytes but also various epithelial cells, with direct involvement in the formation of NPCs and GCs. Already malignantly transformed EBV-negative cell lines, e.g., lymphoma cells, were deliberately avoided as transfection models because they could already have individually different accumulated defects in the expression of certain tumor-immunologically relevant genes/pathways or altered RNA splicing and methylation patterns, as well as accumulated genetic mutations. In order to achieve very high expression levels when overexpressing isolated viral factors, such as an EBV miR, while maintaining high transfection efficiency per se and a non-malignantly transformed cell line as a transfection model with intact gene expression and cell signaling, HEK293T cells were applied, as in other comparable analogous studies, even though HEK293T cells themselves have no context to EBV. The extension of the applied transfection model to also include healthy primary lymphocytes and corresponding healthy epithelial cells, e.g., of the oropharynx, would increase the significance of this study, but this was not possible for several reasons.

For the first time, all EBV miRs were quantified in selected EBV-positive cell lines; the most highly expressed ones were identified, and respective overexpression model systems were generated. Comparative transcriptome analyses were performed, offering novel insights into the molecular mechanisms of EBV-induced malignant transformation and immune evasion-highlighting a particularly relevant impact, especially of ebv-miR-BHRF1-1. Furthermore, the expression values of these EBV miRs, as well as the identified dysregulated tumor-immunological pathways, might exert certain potential as putative tumor disease-related molecular markers and might represent interesting targets for balancing molecular anti-tumor therapies.

## Data Availability

Dataset available on request from the authors.

## Supplementary Materials

The following supporting information can be downloaded at: https://www.mdpi.com/article/10.3390/ijms25147838/s1.

## Abbreviations

BL: Burkitt’s lymphoma; bp, base pairs; DNA, deoxyribonucleic acid; ds, double-stranded; EBV, Epstein–Barr virus; etc., et cetera; GC, gastric cancer; IM, infectious mononucleosis; LMP, latent membrane proteins; miR, microRNA; NPC, nasopharyngeal carcinoma; nt, nucleotide; UTR, untranslated region.
