# Supplementary material for: Analyzing the Impact of the Highest Expressed Epstein–Barr Virus-Encoded microRNAs on the Host Cell Transcriptome
Source: Int J Mol Sci. 2024 Jul 17;25(14):7838. doi: 10.3390/ijms25147838 (PMC11276978; doi:10.3390/ijms25147838)
Supplement: Supplementary file 1 [file ijms-25-07838-s001.zip › supplemental table s4.pdf]

Table S4

name	sequence (5'-3')	application
pan reverse PCR	GTGCAGGGTCCGAGGT	qPCR
SL BART1-5p	GTCGTATCCAGTGCAGGGTCCGAGGTATTCGCACTGGATACGACCACAGC	cDNA synthesis
fwPCR BART1-5p	GCCCTCTTAGTGGAAGTGACGT	qPCR
SL BART1-3p	GTCGTATCCAGTGCAGGGTCCGAGGTATTCGCACTGGATACGACGACATA	cDNA synthesis
fwPCR BART1-3p	GCCCTAGCACCGCTATCCAC	qPCR
SL BART2-5p	GTCGTATCCAGTGCAGGGTCCGAGGTATTCGCACTGGATACGACGCAAGG	cDNA synthesis
fwPCR BART2-5p	GCCCTATTTTCTGCATTCGC	qPCR
SL BART2-3p	GTCGTATCCAGTGCAGGGTCCGAGGTATTCGCACTGGATACGACTTTATT	cDNA synthesis
fwPCR BART2-3p	GCCCAAGGAGCGATTTGGAGAA	qPCR
SL BART3-5p	GTCGTATCCAGTGCAGGGTCCGAGGTATTCGCACTGGATACGACAGCACA	cDNA synthesis
fwPCR BART3-5p	GCCCACCTAGTGTTAGTGT	qPCR
SL BART3-3p	GTCGTATCCAGTGCAGGGTCCGAGGTATTCGCACTGGATACGACACACCT	cDNA synthesis
fwPCR BART3-3p	GCCCCGCACCACTAGTCACC	qPCR
SL BART4-5p	GTCGTATCCAGTGCAGGGTCCGAGGTATTCGCACTGGATACGACAGCACA	cDNA synthesis
fwPCR BART4-5p	GCCCGACCTGATGCTGCTGG	qPCR
SL BART4-3p	GTCGTATCCAGTGCAGGGTCCGAGGTATTCGCACTGGATACGACACACCT	cDNA synthesis
fwPCR BART4-3p	GCCCCACATCACGTAGGCACC	qPCR
SL BART5-5p	GTCGTATCCAGTGCAGGGTCCGAGGTATTCGCACTGGATACGACCGATGG	cDNA synthesis
fwPCR BART5-5p	GCCCCAAGGTGAATATAGCTGC	qPCR
SL BART5-3p	GTCGTATCCAGTGCAGGGTCCGAGGTATTCGCACTGGATACGACAGGTGA	cDNA synthesis
fwPCR BART5-3p	GCCCGTGGGCCGCTGT	qPCR
SL BART6-5p	GTCGTATCCAGTGCAGGGTCCGAGGTATTCGCACTGGATACGACCCTATG	cDNA synthesis
fwPCR BART6-5p	GCCCTAAGGTTGGTCCAATC	qPCR
SL BART6-3p	GTCGTATCCAGTGCAGGGTCCGAGGTATTCGCACTGGATACGACTCTAAG	cDNA synthesis
fwPCR BART6-3p	GCCCCGGGGATCGGACTAGC	qPCR
SL BART7-5p	GTCGTATCCAGTGCAGGGTCCGAGGTATTCGCACTGGATACGACTGTTTC	cDNA synthesis
fwPCR BART7-5p	GCCCCCTGGACCTTGACTAT	qPCR
SL BART7-3p	GTCGTATCCAGTGCAGGGTCCGAGGTATTCGCACTGGATACGACCCCTGG	cDNA synthesis
fwPCR BART7-3p	GCCCCATCATAGTCCAGTGT	qPCR
SL BART8-5p	GTCGTATCCAGTGCAGGGTCCGAGGTATTCGCACTGGATACGACCTGTAC	cDNA synthesis
fwPCR BART8-5p	GCCCTACGGTTTCCTAGATT	qPCR

SL BART8-3p	GTCGTATCCAGTGCAGGGTCCGAGGTATTCGCACTGGATACGACTCTACG	cDNA synthesis
fwPCR BART8-3p	GCCCGTCACAATCTATGGGGT	qPCR
SL BART9-5p	GTCGTATCCAGTGCAGGGTCCGAGGTATTCGCACTGGATACGACGTTTCC	cDNA synthesis
fwPCR BART9-5p	GCCCTACTGGACCCTGAATT	qPCR
SL BART9-3p	GTCGTATCCAGTGCAGGGTCCGAGGTATTCGCACTGGATACGACACTACG	cDNA synthesis
fwPCR BART9-3p	GCCCTAACACTTCATGGGTCC	qPCR
SL BART10-5p	GTCGTATCCAGTGCAGGGTCCGAGGTATTCGCACTGGATACGACTGTACA	cDNA synthesis
fwPCR BART10-5p	GCCCGCCACCTCTTTGGTTC	qPCR
SL BART10-3p	GTCGTATCCAGTGCAGGGTCCGAGGTATTCGCACTGGATACGACACAGCC	cDNA synthesis
fwPCR BART10-3p	GCCCTACATAACCATGGAGTT	qPCR
SL BART11-5p	GTCGTATCCAGTGCAGGGTCCGAGGTATTCGCACTGGATACGACCAACTA	cDNA synthesis
fwPCR BART11-5p	GCCCTCAGACAGTTTGGTGCGC	qPCR
SL BART11-3p	GTCGTATCCAGTGCAGGGTCCGAGGTATTCGCACTGGATACGACGGCAGT	cDNA synthesis
fwPCR BART11-3p	GCCCACGCACACCAGGCTG	qPCR
SL BART12	GTCGTATCCAGTGCAGGGTCCGAGGTATTCGCACTGGATACGACAACCAC	cDNA synthesis
fwPCR BART12	GCCCTCCTGTGGTGTTTGGT	qPCR
SL BART13-5p	GTCGTATCCAGTGCAGGGTCCGAGGTATTCGCACTGGATACGACCTGTAC	cDNA synthesis
fwPCR BART13-5p	GCCCAACCGGCTCGTGGCTC	qPCR
SL BART13-3p	GTCGTATCCAGTGCAGGGTCCGAGGTATTCGCACTGGATACGACTCAGCC	cDNA synthesis
fwPCR BART13-3p	GCCCTGTAACTTGCCAGGGAC	qPCR
SL BART14-5p	GTCGTATCCAGTGCAGGGTCCGAGGTATTCGCACTGGATACGACTGTAAA	cDNA synthesis
fwPCR BART14-5p	GCCCTACCCTACGCTGCCGA	qPCR
SL BART14-3p	GTCGTATCCAGTGCAGGGTCCGAGGTATTCGCACTGGATACGACATCCCT	cDNA synthesis
fwPCR BART14-3p	GCCCTAAATGCTGCAGTAGT	qPCR
SL BART15	GTCGTATCCAGTGCAGGGTCCGAGGTATTCGCACTGGATACGACTCAAGG	cDNA synthesis
fwPCR BART15	GCCCGTCAGTGGTTTTGTTT	qPCR
SL BART16	GTCGTATCCAGTGCAGGGTCCGAGGTATTCGCACTGGATACGACAGAGCA	cDNA synthesis
fwPCR BART16	GCCCTTAGATAGAGTGGGTGTG	qPCR
SL BART17-5p	GTCGTATCCAGTGCAGGGTCCGAGGTATTCGCACTGGATACGACCTTGTA	cDNA synthesis
fwPCR BART17-5p	GCCCTAAGAGGACGCAGGCA	qPCR
SL BART17-3p	GTCGTATCCAGTGCAGGGTCCGAGGTATTCGCACTGGATACGACACTAAG	cDNA synthesis
fwPCR BART17-3p	GCCCTGTATGCCTGGTGTCCC	qPCR
SL BART18-5p	GTCGTATCCAGTGCAGGGTCCGAGGTATTCGCACTGGATACGACTGTATA	cDNA synthesis

fwPCR BART18-5p	GCCCTCAAGTTCGCACTTCC	qPCR
SL BART18-3p	GTCGTATCCAGTGCAGGGTCCGAGGTATTCGCACTGGATACGACGACGAA	cDNA synthesis
fwPCR BART18-3p	GCCCTATCGGAAGTTTGGGC	qPCR
SL BART19-5p	GTCGTATCCAGTGCAGGGTCCGAGGTATTCGCACTGGATACGACCATGTC	cDNA synthesis
fwPCR BART19-5p	GCCCACATTCCCCGCAAACAT	qPCR
SL BART19-3p	GTCGTATCCAGTGCAGGGTCCGAGGTATTCGCACTGGATACGACAGCATT	cDNA synthesis
fwPCR BART19-3p	GCCCTTTTGTTTGCTTGGG	qPCR
SL BART20-5p	GTCGTATCCAGTGCAGGGTCCGAGGTATTCGCACTGGATACGACGGAATG	cDNA synthesis
fwPCR BART20-5p	GCCCTAGCAGGCATGTCTT	qPCR
SL BART20-3p	GTCGTATCCAGTGCAGGGTCCGAGGTATTCGCACTGGATACGACGGTAAC	cDNA synthesis
fwPCR BART20-3p	GCCCCATGAAGGCACAGCCT	qPCR
SL BART21-5p	GTCGTATCCAGTGCAGGGTCCGAGGTATTCGCACTGGATACGACGTTAGT	cDNA synthesis
fwPCR BART21-5p	GCCCTCACTAGTGAAGGCA	qPCR
SL BART21-3p	GTCGTATCCAGTGCAGGGTCCGAGGTATTCGCACTGGATACGACAAACAC	cDNA synthesis
fwPCR BART21-3p	GCCCCTAGTTGTGCCCACTG	qPCR
SL BART22	GTCGTATCCAGTGCAGGGTCCGAGGTATTCGCACTGGATACGACACTACT	cDNA synthesis
fwPCR BART22	GCCCTTACAAAGTCATGGTCT	qPCR
SL BHRF1-1	GTCGTATCCAGTGCAGGGTCCGAGGTATTCGCACTGGATACGACAACTCC	cDNA synthesis
fwPCR BHRF1-1	GCCCTAACCTGATCAGCCCC	qPCR
SL BHRF1-2-5p	GTCGTATCCAGTGCAGGGTCCGAGGTATTCGCACTGGATACGACGCTATC	cDNA synthesis
fwPCR BHRF1-2-5p	GCCCAAATTCTGTTGCAGCA	qPCR
SL BHRF1-2-3p	GTCGTATCCAGTGCAGGGTCCGAGGTATTCGCACTGGATACGACTCAATT	cDNA synthesis
fwPCR BHRF1-2-3p	GCCCTATCTTTTGCGGCAGA	qPCR
SL BHRF1-3	GTCGTATCCAGTGCAGGGTCCGAGGTATTCGCACTGGATACGACTGTGCT	cDNA synthesis
fwPCR BHRF1-3	GCCCTAACGGGAAGTGTGTA	qPCR
SL hsa-miR3960	GTCGTATCCAGTGCAGGGTCCGAGGTATTCGCACTGGATACGACCCCCCG	cDNA synthesis
fwPCR hsa-miR3960	GCCCGGCGGCGGCGGAGGC	qPCR
cloneBHRF1-1fw	AAAGAATTCCCCGCCTTTAGGAAGCAC	cloning
cloneBHRF1-rev	AAAGGATCCCGTTCCAGATGCACCCAA	cloning
cloneBART4fw	AAAGAATTCCCAGGTGTCACCGGAGGC	cloning
cloneBART4rev	AAAGGATCCCCCTTCTCCGGTGAGGAG	cloning
cloneBART1fw	AAACTCGAGGGGGGTCTTAGTGGAAGTGA	cloning
cloneBART1rev	AAAGAATTCCGGGCGAGACATAGTGGATAG	cloning
cloneBART17fw	AAAGAATTCGTTGAACAGGATGTGGCACCC	cloning

cloneBART17rev	AAAGGATCCGCTACCTAGGCCTGCGTCCC	cloning
