# Supplementary figures and images for: Analyzing the Impact of the Highest Expressed Epstein–Barr Virus-Encoded microRNAs on the Host Cell Transcriptome
Source: Int J Mol Sci. 2024 Jul 17;25(14):7838. doi: 10.3390/ijms25147838 (PMC11276978; doi:10.3390/ijms25147838)

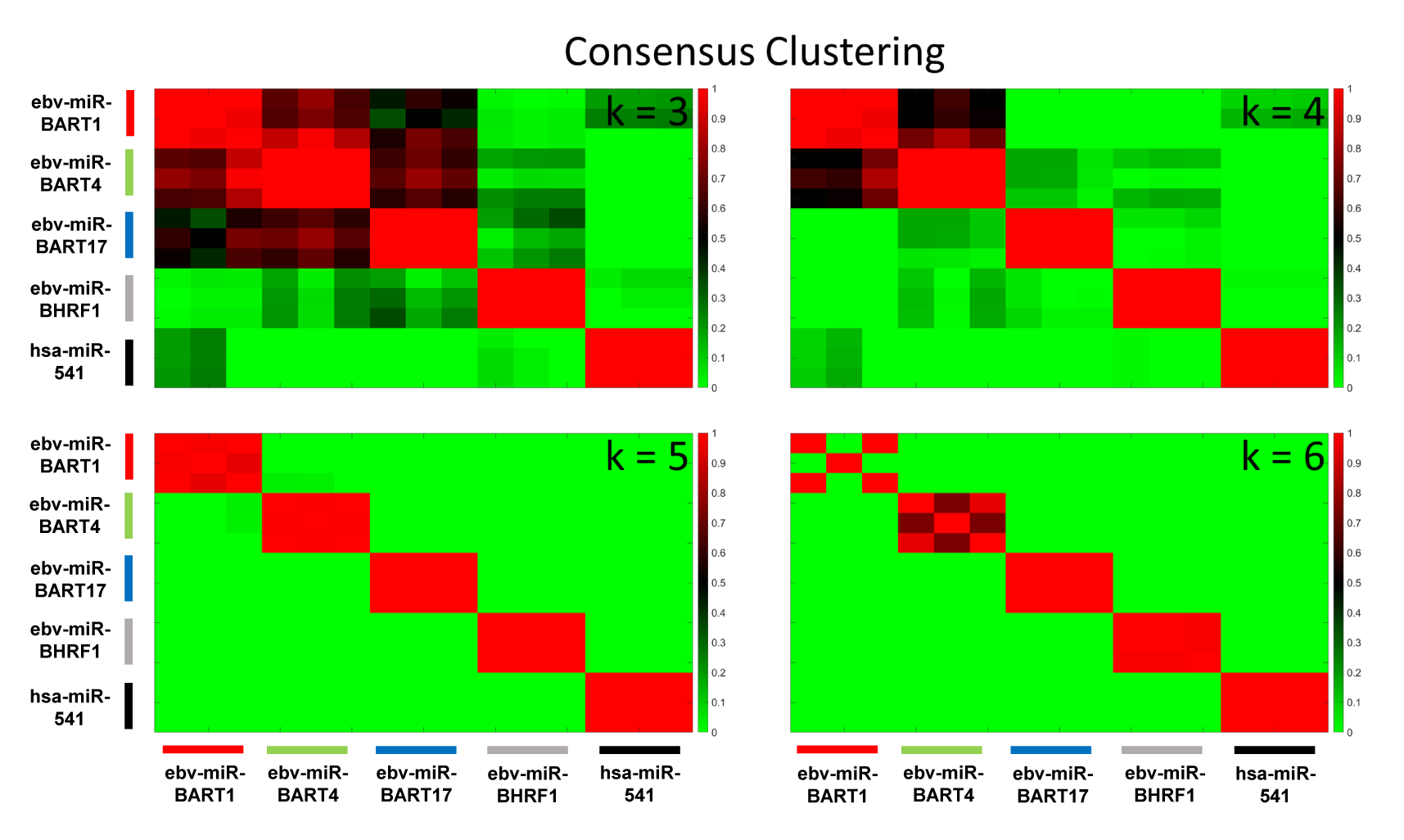

Supplement: Supplementary file 1 [file ijms-25-07838-s001.zip › Supplemental Figure 1.tif]

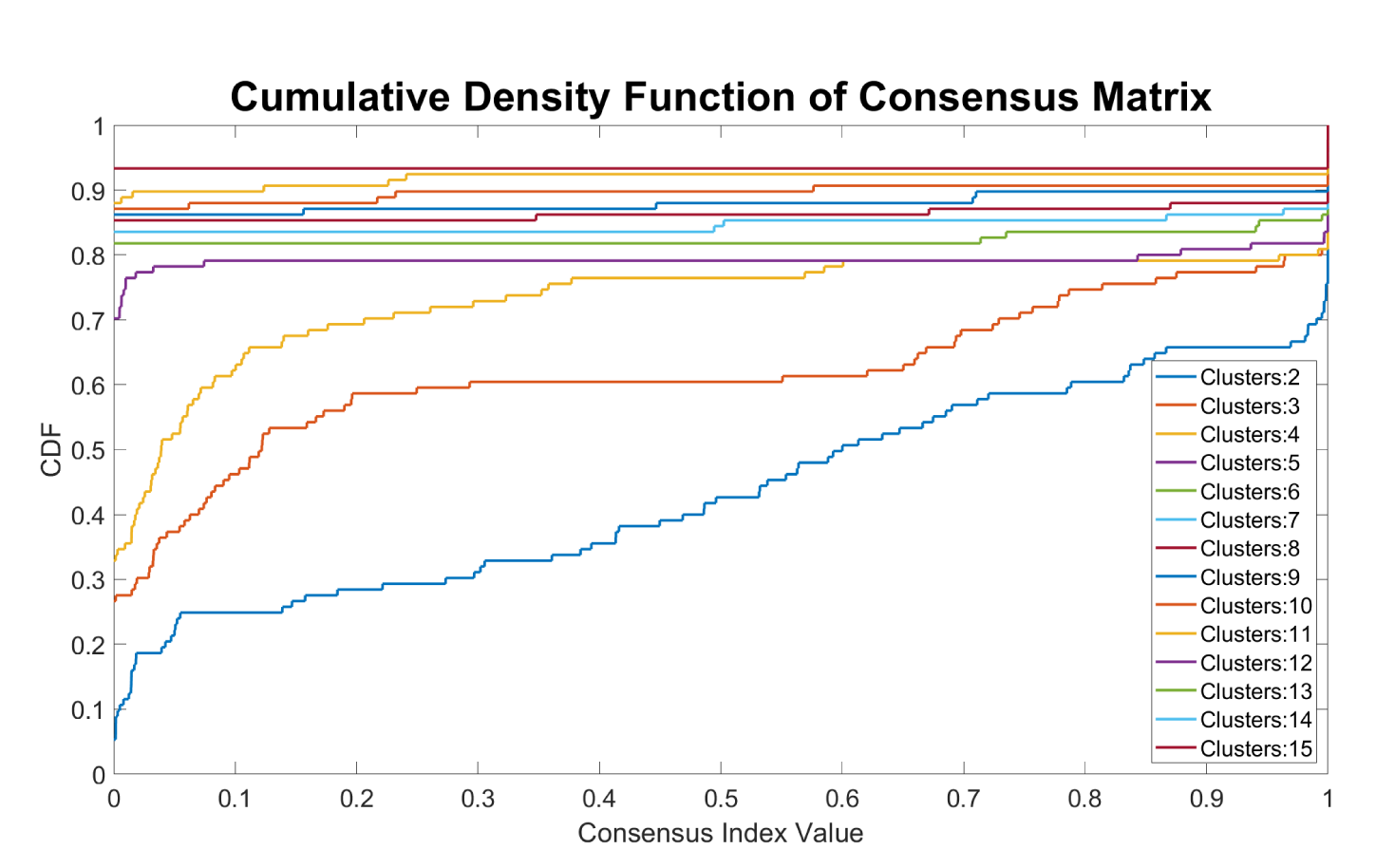

Supplement: Supplementary file 1 [file ijms-25-07838-s001.zip › Supplemental Figure 2.tif]
